# Highly accelerated knee magnetic resonance imaging using deep neural network (DNN)–based reconstruction: prospective, multi-reader, multi-vendor study

**DOI:** 10.1038/s41598-023-44248-7

**Published:** 2023-10-12

**Authors:** Joohee Lee, Min Jung, Jiwoo Park, Sungjun Kim, Yunjin Im, Nim Lee, Ho-Taek Song, Young Han Lee

**Affiliations:** 1 Department of Radiology, Research Institute of Radiological Science, and Center for Clinical Imaging Data Science (CCIDS), Yonsei University College of Medicine, 50-1 Yonsei-ro, Seodaemun-gu, Seoul, 03722 Korea; 2 Department of Orthopaedic Surgery, Yonsei University College of Medicine, Seoul, Korea

**Keywords:** Magnetic resonance imaging, Musculoskeletal system

## Abstract

In this prospective, multi-reader, multi-vendor study, we evaluated the performance of a commercially available deep neural network (DNN)–based MR image reconstruction in enabling accelerated 2D fast spin-echo (FSE) knee imaging. Forty-five subjects were prospectively enrolled and randomly divided into three 3T MRIs. Conventional 2D FSE and accelerated 2D FSE sequences were acquired for each subject, and the accelerated FSE images were reconstructed and enhanced with DNN–based reconstruction software (FSE-DNN). Quantitative assessments and diagnostic performances were independently evaluated by three musculoskeletal radiologists. For statistical analyses, paired *t*-tests, and Pearson’s correlation were used for image quality comparison and inter-reader agreements. Accelerated FSE-DNN reduced scan times by 41.0% on average. FSE-DNN showed better SNR and CNR (*p* < 0.001). Overall image quality of FSE-DNN was comparable (*p* > 0.05), and diagnostic performances of FSE-DNN showed comparable lesion detection. Two of cartilage lesions were under-graded or over-graded (n = 2) while there was no significant difference in other image sets (n = 43). Overall inter-reader agreement between FSE-conventional and FSE-DNN showed good agreement (R^2^ = 0.76; *p* < 0.001). In conclusion, DNN-based reconstruction can be applied to accelerated knee imaging in multi-vendor MRI scanners, with reduced scan time and comparable image quality. This study suggests the potential for DNN-accelerated knee MRI in clinical practice.

## Introduction

It is reported that about 25% of adults experience knee pain, resulting in limitations to their functional capabilities and mobility, and causing a negative impact on their quality of life, and the prevalence of knee pain has shown an upward trend over time, regardless of age^1^. Magnetic resonance imaging (MRI) plays an important role in evaluating internal derangements in patients with knee pain^2^. Common indications of knee MRI are trauma, overuse, degeneration, and knee pain. Commonly used knee MRI protocols are parallel image based MR sequences with multi-channel phased array coil^3,4^. Typical sequences are triplane fat-suppressed fluid-sensitive, sagittal PD-weighted, and coronal or axial T1-weighted image with 15–25 min scan time. 3D sequence or deep learning reconstructions may be added^5^. Common MRI protocols consisting of four to five separately acquired 2D fast spin-echo (FSE) or 2D turbo spin-echo (TSE) pulse sequences are commonly used as a standard in clinical practice, providing excellent tissue contrast and high spatial resolution, enabling good assessment of meniscal, ligamentous, and cartilaginous injuries^6^. Accurate and noninvasive imaging evaluation requires three planes of axial, coronal, and sagittal fat-saturated proton density-weighted or intermediate-weighted images, which require repetitive scans with relatively long scan times. Accelerated MRI is essential in knee imaging because patients with knee pain tend to move, causing motion artifacts, especially when scan time is prolonged. Long scan time of MRI scans can result in reduced productivity per MRI scanner and elevated MRI cost^7^. Implementing accelerated MRI techniques can alleviate patient discomfort and enhance the cost-effectiveness of the process.

Recent advances in various accelerated imaging methods have shown the feasibility of accelerated knee MRI, in some cases enabling a 5-min knee imaging protocol^8–10^. Parallel imaging (PI) is one approach to accelerate MRI data acquisition, and it is based on the principle of acquiring spatial encoding data from overlapping phased-array coil elements that sample the MR signal in parallel^11^. Although disadvantages of PI include reduced signal-to-noise ratio (SNR), aliasing, and reconstruction-related artifacts, acceleration with PI allows for rapid imaging due to the advancement of multi-channel phased-array coil technology^12^. In the knee, 2D FSE with PI has been widely utilized for routine 2D FSE protocols^9^. However, PI acceleration factors higher than 2 cannot be reliably achieved in clinical settings without compromising image quality^13^. Compressed sensing (CS) was developed on the premise of reconstructing an image from an under-sampled *k*-space, since the number of data segments in the *k*-space is a direct determinant of image acquisition time^14^. The combination of CS and PI allows even faster imaging, with the resultant image quality deemed acceptable^15^. However, they require a high computational burden during the image reconstruction process with long iteration times, limiting their use in routine clinical practice.

Recently, deep neural network (DNN)–based MRI reconstructions have been proposed, showing great potential to reduce MRI acquisition time^16,17^. Deep learning-based MRI reconstruction techniques have been approved and are being evaluated in clinical practice^18^. Currently, the software requires to be evaluated and monitored from its premarket development to post-market performance in real-world radiology^19^. However, to date, there has been only a few multi-vendor studies^20–22^ that evaluated the image quality and performances with commercially available DNN–based magnetic resonance imaging (MRI) reconstruction.

The purpose of this prospective, multi-reader, multi-vendor study was to evaluate the performance of commercially available DNN-based MR image reconstruction software in enabling accelerated 2D FSE knee imaging in a clinical environment. We hypothesized that highly accelerated 2D FSE knee imaging combined with DNN–based reconstruction would allow a decrease in scan time while yielding comparable image quality and diagnostic performance for ligamentous, meniscal, and cartilaginous lesions against conventional 2D FSE knee MRI.

## Materials and methods

AIRS Medical provided financial support for this prospective study. The authors had control of the data and the information submitted for publication.

### Study population

This prospective study from a single tertiary center was approved by the Institutional Review Board of Yonsei University’s Health System (IRB No: 1-2022-0017). Written informed consent was obtained from all enrolled participants. Our study complied with both the Declaration of Helsinki and the Health Insurance Portability and Accountability Act.

**Figure 1:**
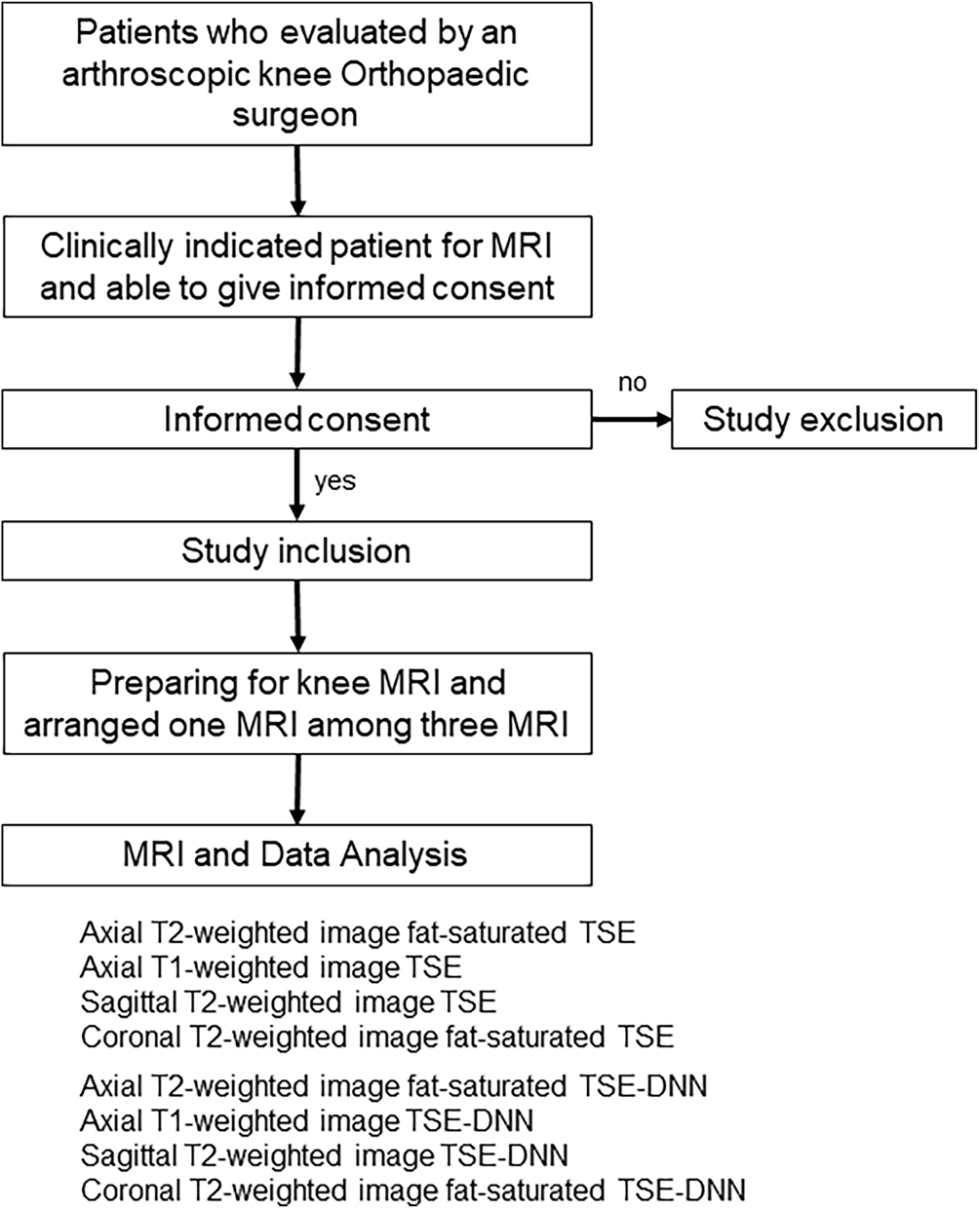
Flow chart for prospective study enrollment to evaluate conventional FSE (FSE-conventional) and accelerated MR sequences with DNN reconstruction (FSE-DNN).

Study recruitment commenced from August 2022 to October 2022. Inclusion criteria were: (1) clinically indicated patient for knee MRI; (2) an agreement to participate in DNN accelerated knee MRI; (3) age of 30 years or older; (4) the ability to position the knee in MRI; and (5) symptomatic knees associated with pain and dysfunction knee. Exclusion criteria consisted of (1) orthopedic implants in the knee region and (2) other general contraindications for MRI. The flow chart for prospective study enrollment is shown in Fig. 1.

### MR imaging protocol

All enrolled patients were randomly assigned to MRI exams with three different 3.0 T MR scanners, and they underwent knee MRI with both conventional and DNN-accelerated MRI sequences: axial fat-saturated T2-weighted image, coronal fat-saturated T2-weighted image, sagittal T2-weighted image, and axial T1-weighted image. MR imaging was performed on three 3.0 T MR scanners (Ellition X, Philips Healthcare, Best, The Netherlands; Prisma Fit, Siemens Healthineers, Erlangen, Germany; Discovery MR750, GE Healthcare, Waukesha, WI, USA) with a dedicated 16-channel knee coil (Philips Healthcare), a dedicated 16-channel knee coil (Siemens Healthineers), and a dedicated 8-channel HD transmit/receive knee coil (GE Healthcare), respectively. Conventional image acquisition utilized knee imaging protocols routinely used at our institution, and the accelerated protocols were achieved by modifying scan time-related MRI parameters. Overall scan time reductions of the DNN-accelerated MRI sequences were 41.0% (43.1%, Philips Healthcare; 38.1%, Siemens Healthineers, 41.72%, GE Healthcare). Accelerated 2D FSE utilized parallel imaging alone such as Sensitivity Encoding (SENSE), Array coil Spatial Sensitivity Encoding (ASSET), and Generalized Autocalibrating Partial Parallel Acquisition (GRAPPA) depending on vendor’s technique. Conventional 2D FSE images utilized combination of compressed sensing (CS) and parallel imaging such as CS-SENSE. A detailed summary of both acquisition methods of MRI parameters is shown in Table 1.

**Table 1:** MRI parameters of FSE-conventional and FSE-DNN in three MRI scanners.

		Philips (Ellition CX)		GE (Discovery MR750)		Siemens (Prisma Fit)	
		FSE-conventional	FSE-DNN	FSE-conventional	FSE-DNN	FSE-conventional	FSE-DNN
Ax T2W FS	TR/TE (ms)	5570/60	5569/60	5193/69	5193/69	4230/63	4230/63
	FOV (cm)	14	14	14	14	14	14
	Matrix	320 × 320	320 × 239	384 × 320	384 × 320	448/403	448/417
	Acceleration	CS-SENSE 2	SENSE 3	ASSET 1	ASSET 2	NEX 2	NEX 1
	Scan time (sec)	108	57	217	111	156	97
		Time reduction	47.22%	Time reduction	48.85%	Time reduction	37.82%
Ax T1W	TR/TE (ms)	636/15	630/15	650/12.382	650/12.376	520/15	520/15
	FOV (cm)	14	14	14	14	14	14
	Matrix	320 × 320	320 × 239	512 × 320	512 × 320	640/448	640/448
	Acceleration	CS-SENSE 2	SENSE 3	ASSET 1	ASSET 2	GRAPPA 2	GRAPPA 3
	Scan time (sec)	51	27	219	114	180	105
		Time reduction	47.06%	Time reduction	47.95%	Time reduction	41.67%
Sag T2W	TR/TE (ms)	3590/100	3590/100	3265/69	3262/60	3690/86	3690/86
	FOV (cm)	14	14	14	14	14	14
	Matrix	512 × 396	512 × 337	320	224	512 × 461	512 × 476
	Acceleration	CS-SENSE 2	SENSE 3	512 × 320	512 × 224	GRAPPA 2	GRAPPA 3
	Scan time (sec)	156	108	190	139	214	146
		Time reduction	30.77%	Time reduction	26.84%	Time reduction	31.78%
Cor T2W FS	TR/TE (ms)	6855/60	6855/60	6414/72.6	6414/72.63	4520/57	4520/57
	FOV (cm)	14	14	14	14	14	14
	Matrix	320 × 320	320 × 239	384 × 288	384 × 288	384 × 288	284 × 288
	Acceleration	CS-SENSE 2	SENSE 3	ASSET 1	ASSET 2	NEX 2	NEX 1
	Scan time (sec)	135	71	229	130	122	72
		Time reduction	47.41%	Time reduction	43.23%	Time reduction	40.98%
Time reduction	(Per MRI)		43.11%		41.72%		38.06%
Overall time reduction		40.96%					

*Ax T2W FS* Axial T2-weighted fat-saturated FSE, *Ax T1W* Axial T1-weighted FSE, *Sag T2W* Sag T2-weighted FSE, *Cor T2W FS* Cor T2-weighted fat-saturated FSE.

### Deep neural network (DNN)–based image reconstruction

**Figure 2:**
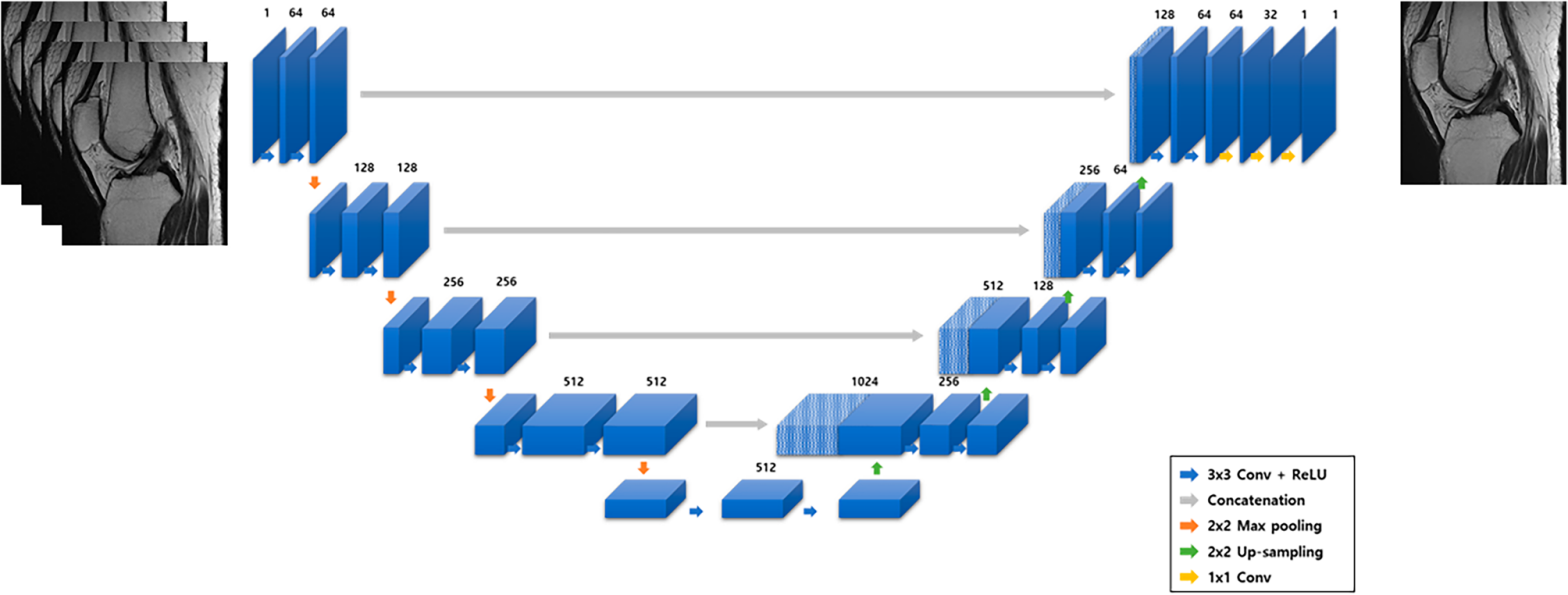
Schematic diagram of FSE-DNN architecture. Input data is DICOM image acquired by accelerated MRI sequence, and output data is enhanced image with denoising.

Commercially available deep neural network (DNN)–based MR image reconstruction software was used to reconstruct the accelerated acquisition images (SwiftMR, v2.0.1.0. AIRS medical, Seoul, Korea). The software algorithm was based on the popular 2D U-net structure^23^ widely used in deep learning architectures in various medical imaging applications. In this model, 18 convolutional blocks, 4 max-pooling layers, 4 up-sampling layers, 4 feature concatenations, and 3 convolutional layers were incorporated in a cascade, with each layer enforcing data consistency. The model was trained and internally validated with 31,865 series and 3540 series of MR images, respectively. The model underwent training using images from the entire body, considering also the musculoskeletal images including the knee. All imaging sequences with different contrasts commonly used in the clinical practice were are included as well. Additionally, the model’s loss function was defined as the structural similarity index (SSIM) between the input and the label image, and the model was optimized with Adam^24^ over 20 epochs using batch size of 4 at a learning rate of 10^–3^, decaying to 10^–4^. The network was trained using four NVIDIA Tesla V100 GPUs with 32 GB memory (NVIDIA Corporation, CA, USA). to evaluate FSE-conventional and accelerated MR sequences with FSE-DNN. Schematic diagram of FSE-DNN architecture was in Fig. 2.

The algorithm includes a deep convolutional neural network (CNN) component that removes noise in the image domain and estimates the truncated high-frequency image data. This pipeline can be applied to 2D and 3D acquisitions in multiple anatomic regions and for various pulse sequences, contrast weightings, field strengths, and coil configurations. The amount of noise reduction could be controlled, owing to the model’s training process incorporating varying levels of noise amount on the input side. For this study, noise reduction level of low (51% reduction) was used because this level had been found to yield the most similar perceived image quality when compared to the standard images.

### Quantitative image quality analysis

For a quantitative comparison of image quality, signal-to-noise ratio (SNR) and contrast-to-noise ratio (CNR) were calculated for all images acquired for this study (conventional, accelerated, and DNN-reconstructed). The femoral bone marrow signal shown in the representative center slice in each image was calculated from a circular region of interest (ROI) placed in the same location for all images, and the mean standard deviation (SD) of the background noise was used for SNR calculation. For CNR, three different imaging plane-landmark combinations were considered—(1) for axial T1-weighted and T2-weighted fat saturated images, marrow-to-muscle signal difference (medial femoral condyle and biceps femoris muscle) and mean SD of background noise were used; (2) for coronal T2-weighted fat saturated images, marrow-to-meniscus signal difference (medial femoral condyle and medial meniscus) and mean SD of background noise were used; (3) for sagittal T2-weighted images, marrow-to-tendon signal difference(distal femur and patellar tendon) and mean SD of background noise were used to calculate the CNR.

### Subjective image quality analysis

Three board-certified musculoskeletal radiologists with six years (J.L.), one year (N.L.), and one year (Y.L.) of subspecialty experience individually assessed the conventional and DNN-reconstructed image sets of the 45 knee MRI, using picture archiving and communication system (PACS) monitors (Totoku, Tokyo, Japan). The radiologists were blind to whether the image was FSE-conventional or FSE-DNN. Each image was assigned a unique random number, and the images were evaluated in a random order. The reviewers scored the images independently based on clarity of overall image quality of anatomical structures, perceived image noise, presence of imaging artifacts using a five-point scale. The clear visibility of the medial meniscus and lateral meniscus; ligamentous structures including anterior cruciate ligament (ACL), posterior cruciate ligament (PCL), and medial collateral ligament (MCL); and cartilage grading were estimated separately.

### Diagnostic performance for ligamentous, meniscal, and cartilaginous lesions

The three radiologists (J.L., N.L., and Y.L.) formed a consensus on grading meniscal lesions, detecting ligamentous lesions, and grading cartilage by reviewing MR images of non-enrolled patients together before evaluation. The radiologists were blinded to the medical records associated with the images acquired for this study. Three radiologists independently evaluated the knee MRI studies for meniscal, tendinous, ligamentous, and osseocartilaginous injuries. For diagnostic performance analysis, all MRI findings and clinical medical records were reviewed to form a reference standard by two additional reviewers (Y.H.L. and M.J.).

The diagnostic criteria for meniscal tears were the presence of abnormal signal intensity within the meniscus extending to the meniscal articular surface and abnormal morphology of the meniscus (0, normal; 1, tear)^25,26^. The diagnostic criteria for ACL are grade 1, minimal lesion with T2 hyperintensities; grade 2, 50% or less injury with T2 hyperintensities; grade 3, complete injury (0, normal; 1, grade 1; 2, grade 2; 3, grade 3)^27^. The diagnostic criteria for PCL tear are an abnormal T2 hyperintensity combined with a discontinuous appearance of the PCL and FCL fibers (0, normal; 1, tear)^28,29^. Intraligamentous ganglion were recorded as grade 1 in ACL and PCL. The diagnostic criteria for MCL tear are grade 1, periligamentous T2 hyperintense with no fiber discontinuity; grade 2, partial discontinuity of the fibers; grade 3, complete ligament (0, normal; 1, grade 1; 2, grade 2; 3, grade 3)^29^.

For qualitative analyses of cartilage grading, the radiologists used the Outerbridge classification system^30,31^ : cartilage grade, Grade 0 = intact cartilage; grade 1 = signal change on T2-weighted MR images; grade 2 = cartilage defect less than 50 percent of the depth; grade 3 = cartilage defect 50% or more of the depth; and grade 4 = full-thickness cartilage defect with exposure of subchondral bone. When multiple cartilage lesions were present, the cartilage lesion with the highest grade was recorded.

### Statistical analysis

Paired *t*-tests were performed to assess the statistical significance of the difference in the quantitative evaluation of SNR and CNR. For the subjective analysis, we calculated the difference in the qualitative image quality score of anatomical structures, perceived image noise, presence of imaging artifacts by using a paired *t*-test, and inter-reader agreement was assessed using Pearson’s correlation. Diagnostic performances of the FSE-DNN were analyzed in terms of sensitivity, specificity, area under curve (AUC), and accuracy. To assess the diagnostic performance of the images in the cartilaginous lesion, the agreements of FSE-conventional and FSE-DNN were assessed using Pearson’s correlation. All statistical analyses were performed in MedCalc (MedCalc Software, Ostend, Belgium) and Microsoft Excel (Microsoft, Redmond, WA, USA). *P*-values < 0.05 were considered statistically significant.

## Results

### Demographic characteristics and scan time reduction

Forty-five patients who underwent this research protocol of knee MRI including routine and accelerated MR pulse sequence were enrolled in three vendors evenly (15 patients for each scanner). The age range of the 45 patients was 30–78 years (mean age ± standard deviation, 53.9 ± 11.8 years). Fourteen patients were male and 31 were female. A total of 45 MRIs of three-vendors were evaluated. Accelerated FSE-DNN reduced scan times by average 41.0% compared to FSE-conventional (GE 41.7%, Philips 43.1%, Siemens 38.1%), respectively.

### Quantitative image quality analysis

SNR and CNR on accelerated FSE images were significantly decreased with scan time reduction such as changing parallel imaging factors and the number of phase encoding steps. FSE-DNN reconstruction software could enhance the SNR and CNR of accelerated FSE image in a short time. FSE-DNN showed statistically better SNR and CNR than convention FSE : SNR ratio were 2.06, 2.23, 2.63, and 2.10 on axial T2-weighted fat-saturated FSE, axial T1-weighted FSE, sagittal T2-weighted FSE, and coronal T2-weighted fat-saturated FSE, respectively ; CNR radio were 2.14, 3.00, 2.81, and 1.83 of marrow-to-muscle on axial T2-weighted fat-saturated FSE, marrow-to-muscle on axial T1-weighted FSE, marrow-to-meniscus on sagittal T2-weighted FSE, and marrow-to-tendon on coronal T2-weighted fat-saturated FSE, respectively. Scan reductions were 44.63%, 45.56%, 29.80%, and 43.87% in axial T2-weighted fat-saturated FSE, axial T1-weighted FSE, sagittal T2-weighted FSE, and coronal T2-weighted fat-saturated FSE, respectively. The image quality analyses with paired t-test results were summarized in Table 2.

**Table 2:** SNR and CNR results of conventional FSE, accelerated FSE without DNN reconstruction, and accelerated FSE with DNN reconstruction (FSE-DNN).

	Philips (Ellition CX)			GE (Discovery MR750)			Siemens (Prisma Fit)			*p*-value
	FSE-conventional	Accelerated FSE	FSE-DNN	FSE-conventional	Accelerated FSE	FSE-DNN	FSE-conventional	Accelerated FSE	FSE-DNN	
SNR										
Ax T2W FS	4.8 ± 1.3	4.3 ± .9	7.7 ± 2.9	14.2 ± 5.4	8.6 ± 4.4	18.4 ± 12.8	2.0 ± 0.2	2.1 ± 0.4	7.3 ± 2.1	< 0.001
Ax T1W	22.8 ± 4.0	13.7 ± 3.5	28.1 ± 6.3	90.9 ± 51.5	57.8 ± 31.9	101.2 ± 53.6	12.2 ± 1.9	8.0 ± 1.3	34.6 ± 6.5	0.002
Sag T2W	22.4 ± 4.4	18.8 ± 3.4	38.9 ± 7.0	33.7 ± 15.5	30.5 ± 20.6	41.9 ± 34.8	9.9 ± 1.7	8.0 ± 1.5	39.3 ± 15.8	< 0.001
Cor T2W FS	5.0 ± 1.7	4.2 ± .9	11. ± 4.3	9.9 ± 3.1	6.7 ± 2.6	11.9 ± 3.6	3.0 ± 0.5	2.6 ± 0.4	12.5 ± 4.2	< 0.001
CNR										
BM-muscle on T2W FS	9.5 ± 6.5	6.0 ± 6.0	10.1 ± 12.0	14.3 ± 10.2	9.5 ± 10.0	17.7 ± 14.1	8.1 ± 3.1	6.5 ± 2.9	37.5 ± 28.2	0.001
BM-muscle on T1W	54.8 ± 16.4	31.7 ± 16.1	65.1 ± 31.6	165.1 ± 76.8	123.1 ± 55	210.9 ± 88.4	31.6 ± 10.3	21.3 ± 7.7	101.2 ± 43.5	< 0.001
BM-tendon on T2W	69.4 ± 22.7	56.3 ± 13.3	112.4 ± 29.1	172.7 ± 120.8	150.6 ± 121.3	194.1 ± 154.2	32.4 ± 7.8	25.0 ± 5.5	118.9 ± 54.6	< 0.001
BM-meniscus on T2W FS	12.1 ± 9.9	8.6 ± 4.6	22.9 ± 18.0	29.2 ± 17.7	15.9 ± 11.8	31.8 ± 22.8	8.3 ± 5.0	6.3 ± 3.9	31.9 ± 27.1	< 0.001

*Ax T2W FS* Axial T2-weighted fat-saturated FSE, *Ax T1W* Axial T1-weighted FSE, *Sag T2W* Sag T2-weighted FSE, *Cor T2W FS* Cor T2-weighted fat-saturated FSE, *p*-values of paired t-test (Accelerated FSE and FSE-DNN).

### Subjective image quality analysis

**Figure 3:**
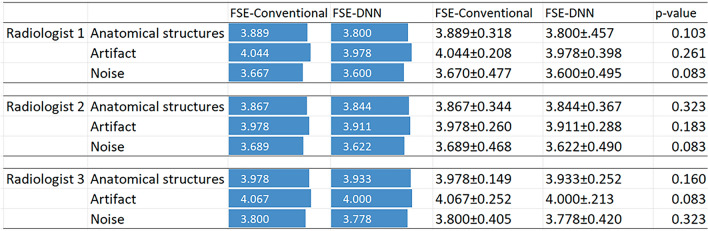
Qualitative evaluation of three radiologists. The comparison between FSE-conventional and FSE-DNN images was shown at overall image quality of anatomical structures, perceived image noise, presence of imaging artifacts using a five-point scale. The average and standard deviation of each value are displayed on right side of each score bar. There are no significantly statistical differences (all, *p*-values > 0.05).

For qualitative evaluation, overall image quality of FSE-DNN was comparable (*p* > 0.05), depending on the reader. Overall image qualities of anatomical structures were better or equal (n = 42/45 in reviewer 1, n = 44/45 in reviewer 2, and n = 43/45 in reviewer 3). Image noise scores were higher or equal (n = 42/45 in reviewer 1 and 2, n = 44/45 in reviewer 3). Imaging artifacts scores were higher or equal (n = 40/45 in reviewer 1, n = 41/45 in reviewer 2, and n = 42/45 in reviewer 3). The qualitative evaluation of three radiologists were displayed in Fig. 3. The comparisons between FSE-conventional and FSE-DNN images were shown at overall image quality of anatomical structures, perceived image noise, presence of imaging artifacts using a five-point scale. The average and standard deviation of each value are displayed on right side of each score bar. There are no significantly statistical differences (all, *p*-values > 0.05). Inter-reader agreements of anatomical structures, perceived image noise, presence of imaging artifacts on FSE and FSE-DNN were fair to moderate correlation (R^2^ = 0.73, 0.31, and 0.89, respectively; all, *p* < 0.001). Inter-reader agreement on FSE and FSE-DNN showed good agreement (R^2^ = 0.76; *p* < 0.001).

**Figure 4:**
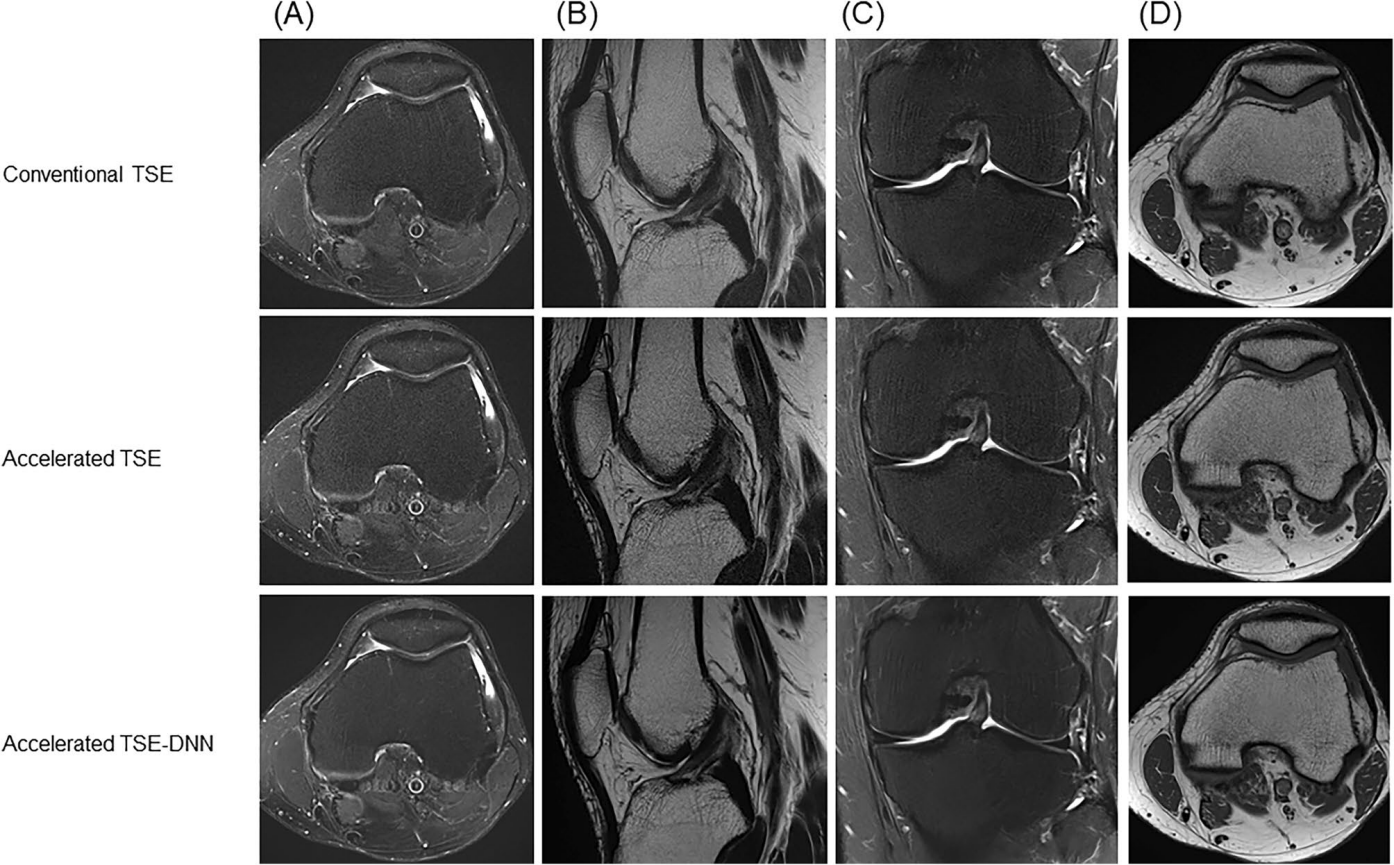
Conventional and reconstructed images of accelerated sequences: a 47-year-old male with knee pain (**A**–**D**). FSE-DNN shows comparable image quality with reduced scan time. The first row represents FSE-conventional images, the second row represents accelerated FSE sequences, and the third row represents DNN-reconstructed images of FSE-DNN. Each column represents the images reconstructed by axial fat-saturated T2-weighted image, sagittal T2-weighted image, coronal fat-saturated T2-weighted image, and axial T1-weighted image.

**Figure 5:**
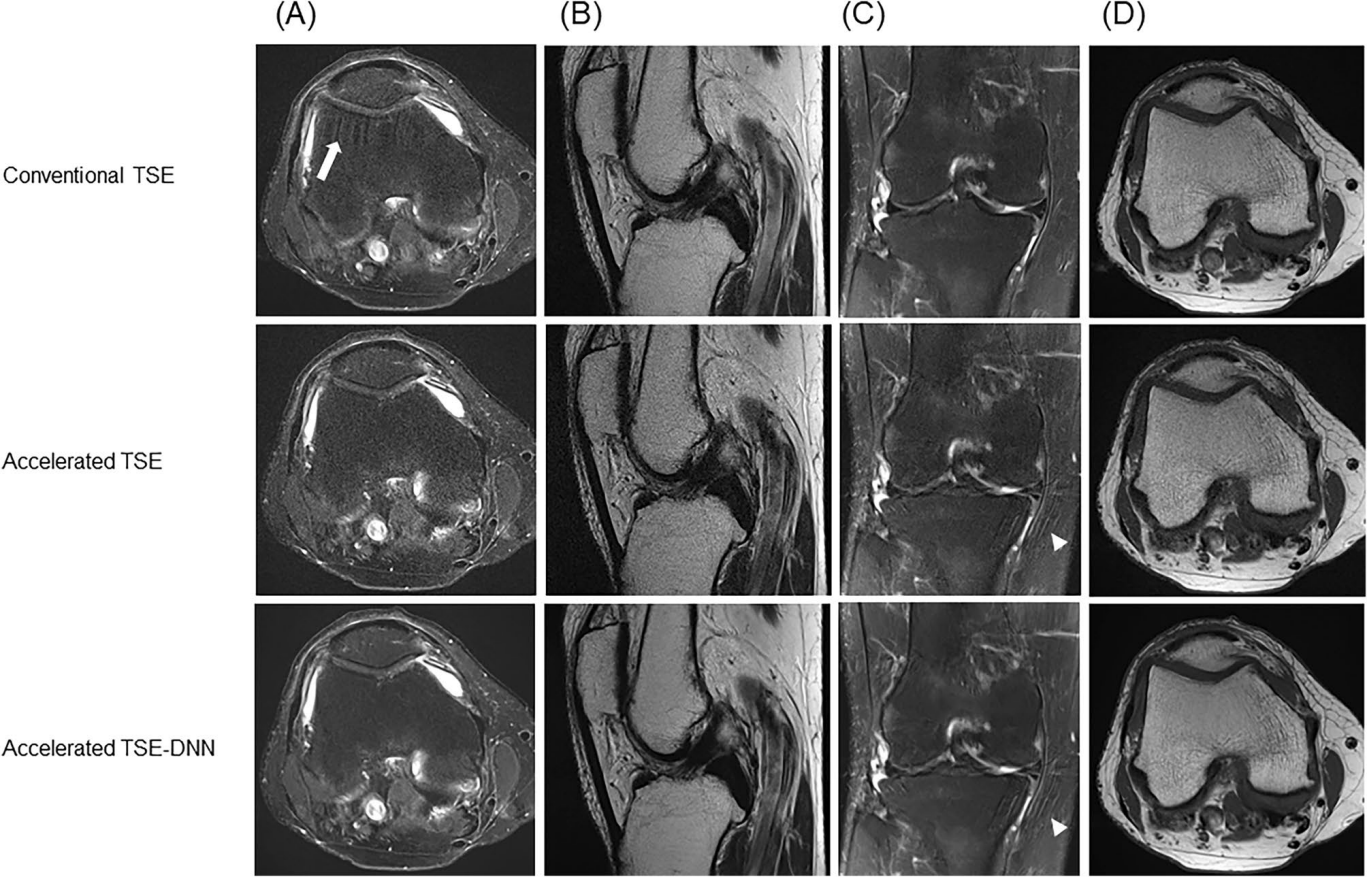
Conventional and reconstructed images of accelerated sequences: a 64-year-old woman with knee pain (**A**–**D**). Motion-related artifacts in conventional FSE (**A** upper, arrow) is not seen on accelerated image (**A** middle), and the image is enhanced on FSE-DNN image (**A** lower). Overall image quality is comparable in both conventional FSE and FSE-DNN images (**B** and **D**). However, parallel imaging artifacts cannot be completely removed in FSE-DNN image (**C** middle and lower, arrowheads). The first row represents conventional FSE images, the second row represents accelerated FSE sequences, and the third row represents DNN-reconstructed images of FSE-DNN. Each column represents the images reconstructed by axial fat-saturated T2-weighted image, sagittal T2-weighted image, coronal fat-saturated T2-weighted image, and axial T1-weighted image.

Representative images for comparable image quality are shown in Fig. 4. The accelerated image exhibited fewer motion-related artifacts, while the TSE-DNN image displayed improved image quality. However, certain artifacts from parallel imaging persisted in the TSE-DNN image, as depicted in Fig. 5.

### Diagnostic performance for ligamentous, meniscal, and cartilaginous lesions

**Figure 6:**
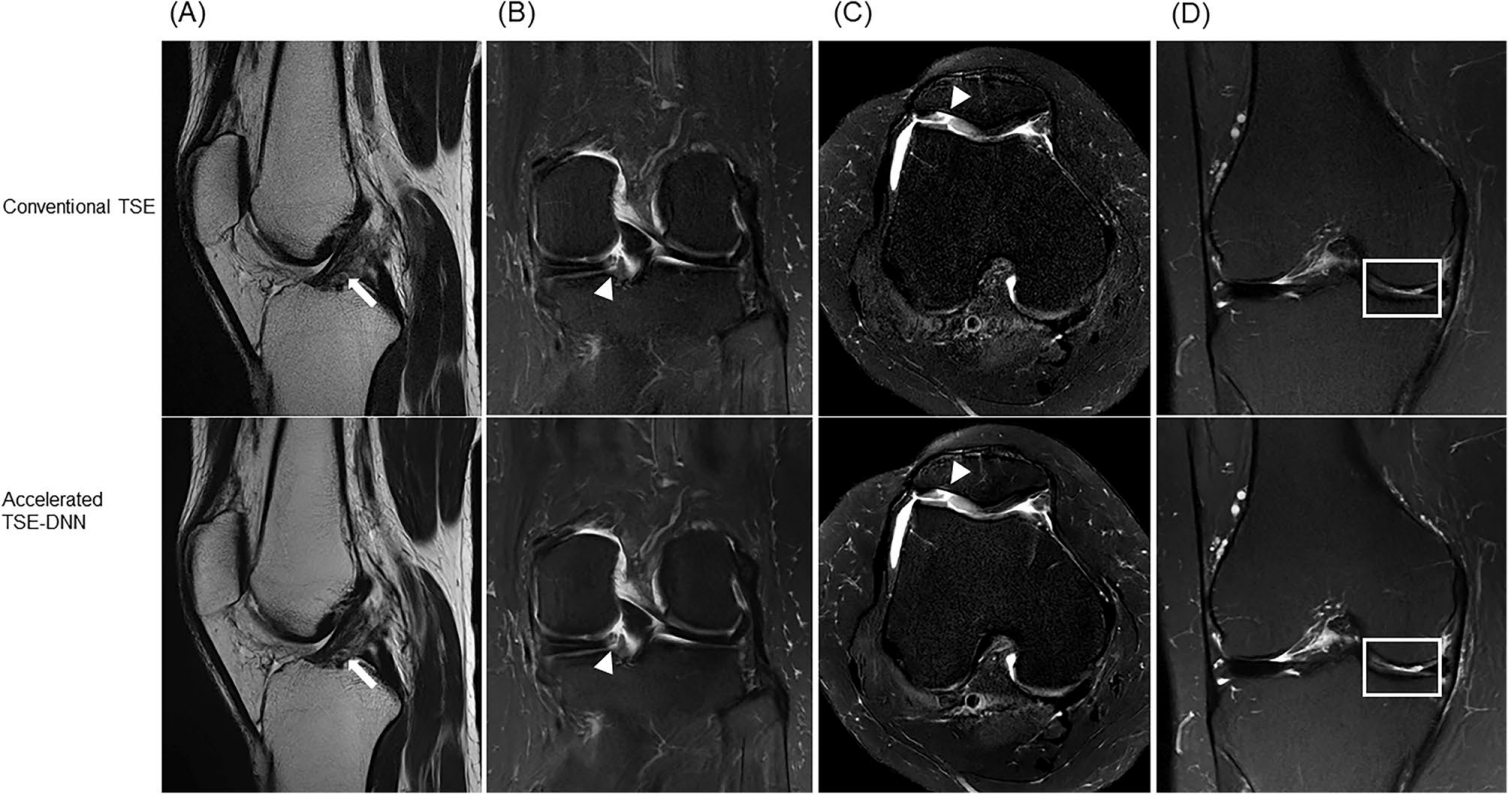
Lesion detection and diagnostic performance in conventional FSE and reconstructed images of accelerated sequences (FSE-DNN). (**A**) A 50-year-old female with knee pain. Mucoid degeneration of ACL (arrows) is shown both conventional FSE (upper **A**) and FSE-DNN images (lower **A**). (**B**) A 60-year-old female with knee pain. Medial meniscal posterior root tear is nicely shown in both images (arrowheads). (**C** and **D**) A 58-year-old female and 58-year-old female with knee pain. Cartilage fissuring (arrowheads) and cartilage flaring (boxes) are well delineated in accelerated FSE-DNN images.

**Figure 7:**
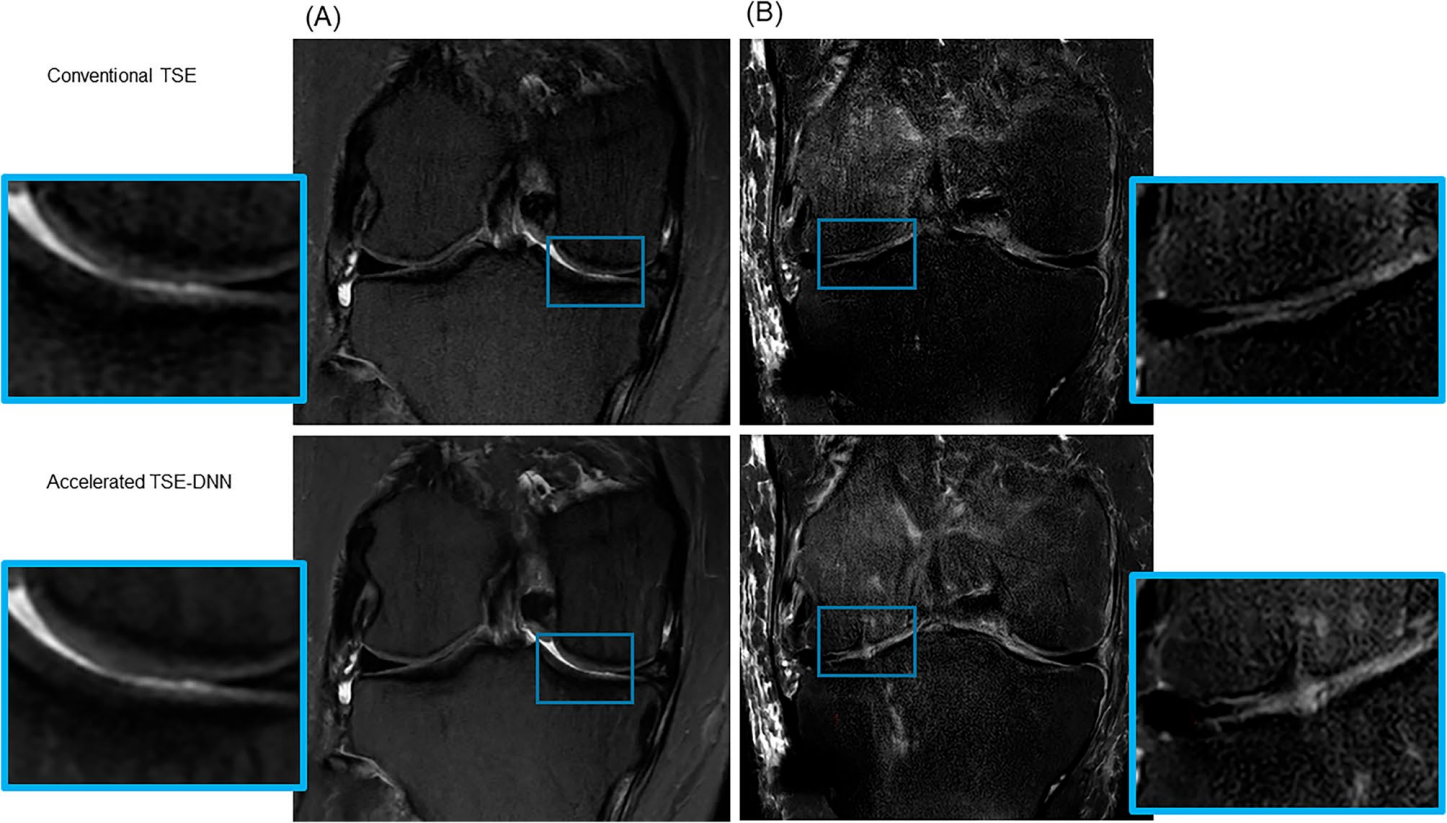
Cartilage grade on conventional and reconstructed images of accelerated sequences. (**A**) A 54-year-old female with knee pain. Cartilage fissuring is shown in medial femoral condyle (upper **A**) while the cartilage fissuring is smoothened, showing under-grade chondromalacia on 2D FSE-DNN image (lower **A**). (**B**) A 41-year-old male with knee pain. Cartilage signal changes without significant defect in lateral tibial plateau (upper **B**) while the cartilage showed T2 high signal intensity defects on 2D FSE-DNN image, showing over-graded cartilage (lower **B**).

All FSE-conv and FSE-DNN images were rated of lesion detection by three interpreting musculoskeletal radiologists. In evaluation of lesion detection, the diagnostic performances of FSE-DNN showed comparable results in ligamentous, meniscal, and cartilaginous lesions (Table 3, Fig. 6). Two of cartilage lesions was under-graded or over-graded (n = 2) while there was no significant difference in other image sets (n = 43). Representative imaging examples for cartilage under-grading or over-grading are shown in Fig. 7.

**Table 3:** Diagnostic performance of DNN-based reconstruction of FSE-DNN.

		MM	LM	ACL	PCL	MCL	FCL	BM	Cartilage
Radiologist 1	Sen (%)	100.00 (76.84–100.00)	100.00 (39.76–100.00)	100.00 (39.76–100.00)	100.00 (15.81–100.00)	100.00 (47.82–100.00)	100.00 (15.81–100.00)	100.00 (87.66–100.00)	Pearson correlation 0.98 (*p* < 0.001)
	Spe (%)	100.00 (88.78–100.00)	100.00 (91.40–100.00)	100.00 (91.40–100.00)	93.02 (80.94–98.54)	100.00 (91.19–100.00)	100.00 (91.78–100.00)	94.12 (71.31–99.85)	
	AUC	1.00 (0.92–1.00)	1.00 (0.92–1.00)	1.00 (0.92–1.00)	0.97 (0.86–1.00)	1.00 (0.92–1.00)	1.00 (0.92–1.00)	0.971 (0.87–1.00)	
	Acc (%)	100.00 (92.13–100.00)	100.00 (92.13–100.00)	100.00 (92.13–100.00)	93.33 (81.73–98.60)	100.00 (92.13–100.00)	100.00 (92.13–100.00)	99.78 (88.23–99.94)	
Radiologist 2	Sen (%)	100.00 (76.84–100.00)	100.00 (39.76–100.00)	80.00 (28.36–99.50)	66.67 (9.43–99.16)	80.00 (28.36–99.50)	50.00 (1.26–98.74)	100.00 (87.66–100.00)	Pearson correlation 0.96 (*p* < 0.001)
	Spe (%)	96.77 (83.30–99.92	100.00 (91.40–100.00)	97.50 (86.84–99.94)	92.86 (80.52–98.50)	100.00 (91.19–100.00)	100.00 (91.78–100.00)	100.00 (80.50–100.00)	
	AUC	0.98 (0.89–1.00)	1.00 (0.92–1.00)	0.89 (0.76–0.96)	0.80 (0.65–0.90)	0.90 (0.77–0.97)	0.75 (0.60–0.87)	1.00 (0.92–1.00)	
	Acc (%)	97.78 (88.23–99.94)	100.00 (92.13–100.00)	95.56 (84.85–99.46)	91.11 (78.78–97.53)	97.78 (88.23–99.94)	97.78 (88.23–99.94)	100.00 (92.13–100.00)	
Radiologist 3	Sen (%)	100.00 (76.84–100.00)	100.00 (39.76–100.00)	100.00 (47.82–100.00)	100.00 (15.81–100.00)	100.00 (47.82–100.00)	100.00 (15.81–100.00)	100.00 (87.66–100.00)	Pearson correlation 0.96 (*p* < 0.001)
	Spe (%)	100.00 (88.78–100.00)	100.00 (91.40–100.00)	100.00 (91.19–100.00)	95.35 (84.19–99.43)	100.00 (91.19–100.00)	100.00 (91.78–100.00)	100.00 (80.50–100.00)	
	AUC	1.00 (0.92–1.00)	1.00 (0.92–1.00)	1.00 (0.92–1.00)	0.98 (0.88–0.99)	1.00 (0.92–1.00)	1.00 (0.92–1.00)	1.00 (0.92–1.00)	
	Acc (%)	100.00 (92.13–100.00)	100.00 (92.13–100.00)	100.00 (92.13–100.00)	95.56 (84.85–99.46)	100.00 (92.13–100.00)	100.00 (92.13–100.00)	100.00 (92.13–100.00)	

*MM* medial meniscus, *LM* lateral meniscus, *ACL* anterior cruciate ligament, *PCL* posterior cruciate ligament, *MCL* medial collateral cruciate ligament, *FCL* fibular collateral ligament, *BM* bone marrow, *Sen* Sensitivity, *Spe* Specificity, *AUC* Area under curve, *Acc* Accuracy, confidence intervals or *p*-values in parentheses.

## Discussion

In this post-market multi-vendor study using commercially available DNN-based parallel imaging reconstruction, the FSE-DNN reconstruction of highly accelerated MRI scan reduced acquisition time by an overall 41.0% for a 2D FSE image of the knee MRI. This algorithm is an image-based DNN-reconstruction, which does not require the *k*-space data nor MRI physics-related information such as multi-channel coil geometry. An older model of this software has also shown promising generalizability results in pediatric brain^32^ and in prostate imaging as well^33^, indicating that an image-based approach may enable accelerated MR exams in clinically used routine sequences with relatively simple modifications such as changing parallel imaging factors and the number of phase encoding lines, etc. This software could restore highly accelerated images in a short time, displaying clinically acceptable image quality and comparable diagnostic performance. This can be differentiated from physics-based (*k*-space-based) DNN reconstruction^34^ which is inherently closely associated with data acquisition methods and may require more computational power and time. As a result, image-based algorithms can be relatively easily deployed in a variety of clinical settings with less MR vendor dependency, and they can even be applied retrospectively to image data in the PACS server. However, further investigation is warranted to accurately compare the image qualities of image-based and k-space-based DNN reconstruction methods.

The strength of our study is that it is a prospective, multi-reader, multi-vendor study as a post-market surveillance. We applied the reconstruction algorithm to knee MRI sequences from all three vendors, showing the possibility of application to multi-vendor MRI applications in radiology. With this strength of this FSE-DNN model, this image-based DNN reconstruction can be easily employed in the radiologic workflow of multi-vendor MRI with various MRI parameters. By changing the parallel imaging factor or number of phase encoding steps of the conventional routine MR sequence, which is easily applicable in the clinical MR imaging protocol, this DNN image reconstruction can reduce scan times with non-inferior image quality and comparable diagnostic performance.

In the quantitative evaluation, FSE-DNN reconstructed images showed higher SNR and CNR, corresponding with previous FSE-DNN studies^35,36^ and the same software^32,33^. In subjective qualitative evaluation, FSE-DNN reconstructed images of accelerated FSE images showed non-inferiority against FSE-conventional images in terms of qualitative image quality evaluation. Deep learning reconstruction can be employed in various accelerated imaging techniques^37–39^, such as parallel imaging, compressed sensing, or their combination. In our study, we did not compare the combination of compressed sensing and parallel imaging with parallel imaging alone (e.g., CS-SENSE vs. SENSE or CS-SENSE vs. ASSET). Further study on deep learning reconstruction comparison study on combination of CS and parallel imaging is needed in the future.

In our study, overall image qualities of anatomical structures were better or equal in most cases. By utilizing deep learning reconstruction of MRI, it is possible to reduce scan time and minimize patient movement, resulting in motion-less imaging. However, the artifacts related with parallel imaging can be pronounced, and these artifacts may persist in some patients. This highlights the need for optimized MRI sequences tailored for accelerated MR imaging. Further research in this direction is imperative in the future. This optimization of MRI sequences involves the choice of CS and the parallel imaging factor, and optimized *k-space* trajectories.

In the diagnostic performance of lesion analysis, FSE-DNN reconstructed images showed non-inferiority compared to FSE-conventional images. In our study, no significant difference was observed in the diagnostic performance between FSE-conventional and FSE-DNN images. In cartilage evaluation, FSE-DNN showed under-graded lesion (n = 1) and over-graded lesion (n = 1) in small numbers (n = 2/45) among 45 image sets. However, there is no statistical difference between FSE-DNN reconstructed images and FSE-conventional images in this 45-case study. This under-grade or over-grade of cartilage may have originated from acceleration artifacts and image degradation rather than the DNN- reconstruction in our early clinical validation with routine clinical MRI protocols (Fig. 7). Cartilage under-grading on FSE-DNN could also have been affected by the amount of image denoising. This suggests the need for careful selection of acceleration method and denoising settings for cartilage imaging, which may depend on imaging target structures. Conversely, cartilage could be over-graded from parallel imaging-related artifacts. In an under-graded chondromalacia case, cartilage fissuring was smoothened on FSE-DNN images while cartilage signal was slightly enhanced in an over-graded chondromalacia case. This highlights the necessity for MRI sequence optimization, particularly emphasizing the need for more precise learning when it comes to small structures like cartilage and structures influenced by MR signal intensity. This acceleration optimization could be different depending on the target joint (e.g. a large off-center shoulder and a small extremity hand) and target structures such as ligaments, bone marrow, meniscus, and cartilage. Further study involving a larger number of images is needed to validate this aspect.

There were several limitations of this study as well. First, the acquisition parameter modifications were not the same between the three vendors. For example, compressed sensing is routinely used in only one of the scanners, whereas the other two scanners utilize parallel imaging only. We intended the DNN-based reconstruction application to the current MRI sequences, reflecting the clinical practice. Secondly, we set a denoising level of low (51% reduction) according to a preceding internal study on noise reduction level for knee MRI. However, optimal image reduction level should be further investigated in clinical MRI, which could depend on the scanner, imaging joint or target, and radio-frequency coil. Thirdly, our diagnostic evaluations were not confirmed arthroscopically in all patients. We conducted this study with radiologists’ consensus as a gold standard of diagnostic performance. Despite evaluation in limited number of patients and limited pathologic findings, this prospective study supports the possibility and the generalizability of DNN reconstruction of highly accelerated MRI to reduce the MRI scan time in clinical practice. Fourthly, the measurements for SNR and CNR were conducted by conventional approach. While optimized methods are available for sensitivity map-based parallel imaging^40,41^, it was necessary to adhere to the conventional approach for practical reasons, as multiple acquisitions were challenging to perform on actual patient images. And, in the context of deep learning-based MRI reconstruction, an optimized method has not yet been established. Further research in this area is warranted in the future.

In conclusion, DNN can be applied to accelerated knee imaging in multi-vendor MRI scanners, with reduced scan time and comparable image quality. Cartilage grading could be under- or over-graded while good agreements in ligamentous and meniscal evaluations. Therefore, the readers should be cautious in utilizing in DNN-accelerated MRI for some lesion evaluation. This study suggests the potential for routine MRI protocols applications to DNN-accelerated knee MRI in clinical practice.

## Data Availability

The datasets generated during and/or analyzed during the current study are available from the corresponding author on reasonable request.

## Abbreviations

Acc: Accuracy
ACL: Anterior cruciate ligament
AUC: Area under curve
Ax T1W: Axial T1-weighted images
Ax T2W FS: Axial T2-weighted fat-saturated images
BM: Bone Marrow
CNN: Convolutional neural network
Cor T2W FS: Coronal T2-weighted fat-saturated images
CS: Compressed sensing
DNN: Deep neural network
FCL: Fibular collateral ligament
FSE: Fast spin-echo
HIPAA: Health insurance portability and accountability act
LM: Lateral meniscus
MCL: Medial collateral ligament
MM: Medial meniscus
MRI: Magnetic resonance imaging
PACS: Picture archiving and communication system
PCL: Posterior cruciate ligament
PI: Parallel imaging
ROI: Region of interest
Sag T2W: Sagittal T2-weighted images
SD: Standard deviation
Sen: Sensitivity
SNR: Signal-to-noise ratio
Spe: Specificity
SSIM: Structural similarity index
TSE: Turbo spin-echo
